# 4,4-Difluoro-8-(4-iodo­phen­yl)-1,3,5,7-tetra­methyl-3a-aza-4a-azonia-4-borata-*s*-indacene

**DOI:** 10.1107/S1600536812004072

**Published:** 2012-04-04

**Authors:** Yongling Sun

**Affiliations:** a Department of Biology, Dezhou University, Dezhou 253023, People’s Republic of China

## Abstract

In the title compound, C_19_H_18_BF_2_IN_2_, which is a boron–dipyrromethene (BODIPY) derivative, the BODIPY mean plane forms dihedral angles of 88.95 (4) and 78.21 (3)° with the F/B/F and 4-iodo­phenyl planes, respectively.

## Related literature

For the crystal structures of related boron–dipyrromethene derivatives, see: Zhou (2010 ▶); Chen & Jiang (2011 ▶); Hinkle *et al.* (2011 ▶); Cui *et al.* (2012 ▶).
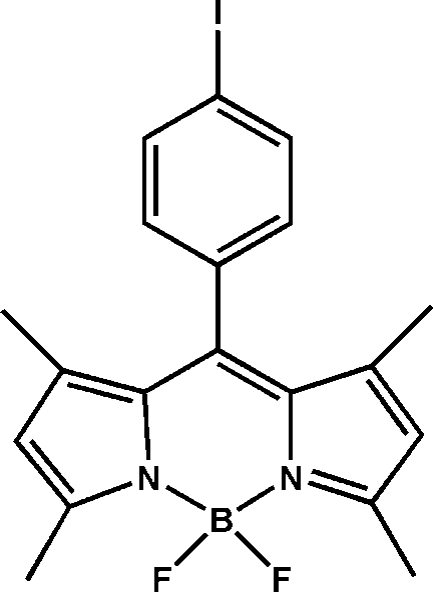


## Experimental

### 

#### Crystal data

C_19_H_18_BF_2_IN_2_
*M* *_r_* = 450.06
Monoclinic, 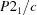

*a* = 12.1004 (3) Å
*b* = 8.1992 (2) Å
*c* = 18.0607 (4) Å
β = 90.577 (3)°
*V* = 1791.77 (8) Å^3^
*Z* = 4
Cu *K*α radiation
μ = 14.24 mm^−1^
*T* = 293 K
0.20 × 0.18 × 0.16 mm

#### Data collection

Bruker SMART 1000 CCD area-detector diffractometer
Absorption correction: multi-scan (*SADABS*; Bruker, 2007 ▶) *T*_min_ = 0.102, *T*_max_ = 0.110
6829 measured reflections
3348 independent reflections
2770 reflections with *I* > 2σ(*I*)
*R*_int_ = 0.044

#### Refinement

*R*[*F* ^2^ > 2σ(*F* ^2^)] = 0.062
*wR*(*F* ^2^) = 0.166
*S* = 1.05
3348 reflections
230 parameters
H-atom parameters constrained
Δρ_max_ = 1.33 e Å^−3^
Δρ_min_ = −1.36 e Å^−3^

### 

Data collection: *SMART* (Bruker, 2007 ▶); cell refinement: *SAINT* (Bruker, 2007 ▶); data reduction: *SAINT*; program(s) used to solve structure: *SHELXS97* (Sheldrick, 2008 ▶); program(s) used to refine structure: *SHELXL97* (Sheldrick, 2008 ▶); molecular graphics: *SHELXTL* (Sheldrick, 2008 ▶); software used to prepare material for publication: *SHELXTL*.

## Supplementary Material

Crystal structure: contains datablock(s) I, global. DOI: 10.1107/S1600536812004072/cv5238sup1.cif

Structure factors: contains datablock(s) I. DOI: 10.1107/S1600536812004072/cv5238Isup2.hkl

Supplementary material file. DOI: 10.1107/S1600536812004072/cv5238Isup3.cml

Additional supplementary materials:  crystallographic information; 3D view; checkCIF report

## Figures and Tables

**Fig. 1.**
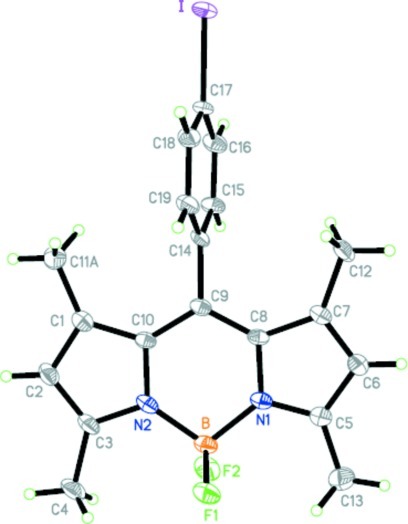
The molecular structure of the title compound. Displacement ellipsoids are drawn at the 30% probability level.

## supplementary crystallographic information

### Comment

In our search for new potential boron-dipyrromethene (BODIPY) fluorescent dyes (Chen & Jiang, 2011), we have obtained the title compound (I). Herewith we report its crystal structure.

In (I) (Fig. 1), all bond lengths and angles are normal in relation to to those observed in the related boron-dipyrromethene derivatives (Zhou, 2010; Hinkle *et al.*, 2011; Cui *et al.*, 2012). The C—C and C—N bond lengths within BODIPY fragment are in the range of 1.371–1.422 and 1.337–1.402 Å, respectively, without any clear distinction between single and double bonds, indicating strongly delocalized π-system. The C_9_BN_2_ fragment is essentially flat, with the maximum deviation from the least-squares mean plane of 0.065 (3) Å. The dihedral angle between the F—B—F plane and the BODIPY mean plane is 88.95 (4)°. Due to the presence of two methyl groups attached to C1 and C7 atoms in BODIPY fragment, the dihedral angles between the BODIPY mean plane and 4-iodophenyl fragment is 78.21 (3)°.

### Experimental

To the mixture of *p*-iodobenzyldehyde (231 mg, 1 mmol) and 2,4-dimethylpyrrole (190 mg, 2.00 mmol) dissolved in CH_2_Cl_2_ (150 ml), one drop of TFA was added. After the resulting mixture was stirred for five hours at room temperature under N_2_ atmosphere, a solution of DDQ (227 mg, 1 mmol) in CH_2_Cl_2_ (60 ml) was added and the reaction mixture was further stirred for another 10 min. After the addition of N, *N*-diisopropylethylamine (DIEA) (2 ml) into the mixture for 15 min, the BF_3_—OEt_2_ (2.0 ml) was added into the reaction mixture and stirring was continued for another 30 min. The resulting mixture was evaporated, and the residue was chromatographed on a silica gel column using CH_2_Cl_2_ as eluent. Repeated chromatography followed by recrystallization from CH_2_Cl_2_ and MeOH gave the target compound as red crystals. Yield: 130 mg, 28.9%. Anal. for C_19_H_18_BF_2_IN_2_: Calc. C, 50.70; H, 4.03; N, 6.22; Found: C, 50.42; H, 4.17; N, 6.31%. The No. of CCDC: 863227.

### Refinement

All H atoms were placed in geometrically idealized positions and treated as riding on their parent atoms, with C—H = 0.93 - 0.96 Å and *U*_iso_(H) > 1.2–1.5 *U*_eq_(C).

### Crystal data

**Table:**

C_19_H_18_BF_2_IN_2_	*F*(000) = 888
*M**_r_* = 450.06	*D*_x_ = 1.668 Mg m^−^^3^
Monoclinic, *P*2_1_/*c*	Cu *K*α radiation, λ = 1.54184 Å
*a* = 12.1004 (3) Å	Cell parameters from 3440 reflections
*b* = 8.1992 (2) Å	µ = 14.24 mm^−^^1^
*c* = 18.0607 (4) Å	*T* = 293 K
β = 90.577 (3)°	Block, red
*V* = 1791.77 (8) Å^3^	0.20 × 0.18 × 0.16 mm
*Z* = 4	

### Data collection

**Table:**

Bruker SMART 1000 CCD area-detector diffractometer	3348 independent reflections
Radiation source: fine-focus sealed tube	2770 reflections with *I* > 2σ(*I*)
Graphite monochromator	*R*_int_ = 0.044
phi and ω scans	θ_max_ = 70.8°, θ_min_ = 4.9°
Absorption correction: multi-scan (*SADABS*; Bruker, 2007)	*h* = −9→14
*T*_min_ = 0.102, *T*_max_ = 0.110	*k* = −10→8
6829 measured reflections	*l* = −21→21

### Refinement

**Table:**

Refinement on *F*^2^	Primary atom site location: structure-invariant direct methods
Least-squares matrix: full	Secondary atom site location: difference Fourier map
*R*[*F*^2^ > 2σ(*F*^2^)] = 0.062	Hydrogen site location: inferred from neighbouring sites
*wR*(*F*^2^) = 0.166	H-atom parameters constrained
*S* = 1.05	*w* = 1/[σ^2^(*F*_o_^2^) + (0.0917*P*)^2^ + 0.3111*P*] where *P* = (*F*_o_^2^ + 2*F*_c_^2^)/3
3348 reflections	(Δ/σ)_max_ = 0.001
230 parameters	Δρ_max_ = 1.33 e Å^−^^3^
0 restraints	Δρ_min_ = −1.36 e Å^−^^3^

### Special details

**Table:**

Geometry. All e.s.d.'s (except the e.s.d. in the dihedral angle between two l.s. planes) are estimated using the full covariance matrix. The cell e.s.d.'s are taken into account individually in the estimation of e.s.d.'s in distances, angles and torsion angles; correlations between e.s.d.'s in cell parameters are only used when they are defined by crystal symmetry. An approximate (isotropic) treatment of cell e.s.d.'s is used for estimating e.s.d.'s involving l.s. planes.
Refinement. Refinement of *F*^2^ against ALL reflections. The weighted *R*-factor *wR* and goodness of fit *S* are based on *F*^2^, conventional *R*-factors *R* are based on *F*, with *F* set to zero for negative *F*^2^. The threshold expression of *F*^2^ > σ(*F*^2^) is used only for calculating *R*-factors(gt) *etc*. and is not relevant to the choice of reflections for refinement. *R*-factors based on *F*^2^ are statistically about twice as large as those based on *F*, and *R*- factors based on ALL data will be even larger.

### Fractional atomic coordinates and isotropic or equivalent isotropic displacement parameters (Å^2^)

**Table:**

	*x*	*y*	*z*	*U*_iso_*/*U*_eq_	
I	0.46758 (4)	−0.22865 (6)	0.54374 (2)	0.0515 (2)	
F1	1.0026 (3)	0.3904 (5)	0.8798 (2)	0.0540 (10)	
N2	0.8395 (4)	0.2246 (6)	0.8934 (3)	0.0334 (11)	
F2	0.8499 (4)	0.5070 (5)	0.9279 (2)	0.0569 (11)	
N1	0.8479 (3)	0.4411 (6)	0.7983 (2)	0.0305 (10)	
C15	0.7292 (4)	0.0148 (7)	0.6658 (3)	0.0345 (13)	
H6	0.8056	0.0233	0.6619	0.041*	
C2	0.8031 (5)	−0.0117 (8)	0.9496 (3)	0.0401 (14)	
H7	0.8028	−0.0933	0.9853	0.048*	
C9	0.7415 (4)	0.1937 (7)	0.7768 (3)	0.0278 (11)	
C1	0.7510 (4)	−0.0199 (7)	0.8812 (3)	0.0332 (12)	
C14	0.6760 (4)	0.0933 (7)	0.7238 (3)	0.0280 (11)	
C10	0.7747 (4)	0.1305 (7)	0.8456 (3)	0.0272 (11)	
C8	0.7754 (4)	0.3485 (7)	0.7541 (3)	0.0273 (11)	
C7	0.7538 (4)	0.4399 (7)	0.6886 (3)	0.0325 (12)	
C13	0.9491 (6)	0.7053 (9)	0.7908 (4)	0.0510 (17)	
H15B	1.0239	0.6742	0.7807	0.077*	
H15C	0.9397	0.7166	0.8432	0.077*	
H15A	0.9331	0.8074	0.7669	0.077*	
C6	0.8157 (5)	0.5794 (8)	0.6943 (3)	0.0390 (13)	
H16	0.8192	0.6614	0.6588	0.047*	
C4	0.9226 (6)	0.2003 (10)	1.0198 (4)	0.0522 (18)	
H17C	0.8915	0.3008	1.0371	0.078*	
H17A	0.9972	0.2187	1.0043	0.078*	
H17B	0.9223	0.1214	1.0591	0.078*	
C17	0.5569 (5)	−0.0896 (7)	0.6206 (3)	0.0357 (13)	
C5	0.8727 (5)	0.5783 (8)	0.7620 (3)	0.0354 (13)	
C18	0.5021 (5)	−0.0124 (8)	0.6784 (3)	0.0370 (13)	
H20	0.4259	−0.0218	0.6826	0.044*	
C16	0.6705 (5)	−0.0756 (8)	0.6139 (3)	0.0389 (14)	
H21	0.7069	−0.1262	0.5750	0.047*	
C19	0.5624 (5)	0.0783 (8)	0.7293 (3)	0.0362 (13)	
H22	0.5260	0.1299	0.7679	0.043*	
C3	0.8559 (5)	0.1382 (8)	0.9563 (3)	0.0384 (14)	
B	0.8873 (5)	0.3956 (9)	0.8772 (4)	0.0346 (14)	
C12	0.6745 (5)	0.4019 (9)	0.6266 (3)	0.0465 (16)	
H25A	0.6999	0.3077	0.6002	0.070*	
H25C	0.6703	0.4933	0.5935	0.070*	
H25B	0.6027	0.3804	0.6465	0.070*	
C11A	0.6824 (5)	−0.1606 (8)	0.8552 (4)	0.0419 (14)	
H1AB	0.6056	−0.1314	0.8567	0.063*	
H1AC	0.6955	−0.2529	0.8868	0.063*	
H1AA	0.7020	−0.1878	0.8053	0.063*	

### Atomic displacement parameters (Å^2^)

**Table:**

	*U*^11^	*U*^22^	*U*^33^	*U*^12^	*U*^13^	*U*^23^
I	0.0601 (4)	0.0609 (4)	0.0331 (3)	−0.0223 (2)	−0.0201 (2)	−0.00197 (17)
F1	0.0302 (17)	0.081 (3)	0.050 (2)	−0.0144 (19)	−0.0154 (16)	0.009 (2)
N2	0.031 (2)	0.048 (3)	0.021 (2)	0.001 (2)	−0.0043 (19)	−0.003 (2)
F2	0.082 (3)	0.057 (2)	0.0314 (18)	−0.001 (2)	−0.0013 (19)	−0.0118 (17)
N1	0.025 (2)	0.039 (3)	0.027 (2)	−0.001 (2)	−0.0030 (17)	−0.006 (2)
C15	0.023 (2)	0.047 (3)	0.034 (3)	0.000 (2)	−0.006 (2)	−0.009 (3)
C2	0.043 (3)	0.048 (4)	0.029 (3)	0.003 (3)	−0.009 (2)	0.008 (3)
C9	0.021 (2)	0.037 (3)	0.026 (3)	0.002 (2)	−0.002 (2)	−0.006 (2)
C1	0.028 (3)	0.042 (3)	0.030 (3)	0.004 (3)	0.000 (2)	−0.001 (2)
C14	0.027 (2)	0.040 (3)	0.017 (2)	−0.003 (2)	−0.0031 (19)	0.001 (2)
C10	0.020 (2)	0.035 (3)	0.027 (2)	0.001 (2)	−0.0028 (19)	−0.005 (2)
C8	0.021 (2)	0.033 (3)	0.028 (2)	0.000 (2)	−0.0051 (19)	−0.007 (2)
C7	0.029 (3)	0.043 (3)	0.026 (2)	0.002 (3)	−0.003 (2)	−0.003 (2)
C13	0.047 (4)	0.051 (4)	0.055 (5)	−0.007 (3)	−0.001 (3)	−0.005 (3)
C6	0.046 (3)	0.042 (3)	0.029 (3)	−0.002 (3)	0.000 (2)	0.001 (2)
C4	0.053 (4)	0.075 (5)	0.029 (3)	−0.011 (4)	−0.016 (3)	0.001 (3)
C17	0.044 (3)	0.037 (3)	0.026 (3)	−0.012 (3)	−0.017 (2)	−0.002 (2)
C5	0.034 (3)	0.039 (3)	0.033 (3)	−0.001 (3)	−0.002 (2)	−0.003 (2)
C18	0.027 (3)	0.048 (4)	0.036 (3)	−0.009 (3)	−0.004 (2)	0.004 (3)
C16	0.034 (3)	0.054 (4)	0.028 (3)	0.001 (3)	0.002 (2)	−0.008 (3)
C19	0.035 (3)	0.049 (3)	0.025 (3)	−0.004 (3)	−0.004 (2)	−0.002 (2)
C3	0.038 (3)	0.055 (4)	0.023 (3)	−0.001 (3)	−0.008 (2)	0.002 (3)
B	0.028 (3)	0.047 (4)	0.028 (3)	−0.006 (3)	−0.002 (2)	−0.007 (3)
C12	0.047 (3)	0.061 (4)	0.032 (3)	−0.007 (3)	−0.015 (3)	0.012 (3)
C11A	0.038 (3)	0.044 (4)	0.044 (3)	0.000 (3)	−0.001 (3)	0.003 (3)

### Geometric parameters (Å, º)

**Table:**

I—C17	2.089 (5)	C7—C12	1.500 (7)
F1—B	1.397 (7)	C13—C5	1.483 (9)
N2—C3	1.352 (8)	C13—H15B	0.9600
N2—C10	1.393 (7)	C13—H15C	0.9600
N2—B	1.546 (9)	C13—H15A	0.9600
F2—B	1.373 (8)	C6—C5	1.399 (8)
N1—C5	1.337 (8)	C6—H16	0.9300
N1—C8	1.403 (6)	C4—C3	1.486 (8)
N1—B	1.544 (8)	C4—H17C	0.9600
C15—C16	1.387 (8)	C4—H17A	0.9600
C15—C14	1.392 (7)	C4—H17B	0.9600
C15—H6	0.9300	C17—C16	1.386 (8)
C2—C1	1.382 (8)	C17—C18	1.394 (8)
C2—C3	1.390 (9)	C18—C19	1.386 (8)
C2—H7	0.9300	C18—H20	0.9300
C9—C8	1.396 (8)	C16—H21	0.9300
C9—C10	1.402 (7)	C19—H22	0.9300
C9—C14	1.485 (7)	C12—H25A	0.9600
C1—C10	1.422 (8)	C12—H25C	0.9600
C1—C11A	1.494 (8)	C12—H25B	0.9600
C14—C19	1.385 (8)	C11A—H1AB	0.9600
C8—C7	1.422 (8)	C11A—H1AC	0.9600
C7—C6	1.370 (9)	C11A—H1AA	0.9600
			
C3—N2—C10	107.9 (5)	H17C—C4—H17A	109.5
C3—N2—B	125.6 (5)	C3—C4—H17B	109.5
C10—N2—B	126.4 (5)	H17C—C4—H17B	109.5
C5—N1—C8	108.6 (4)	H17A—C4—H17B	109.5
C5—N1—B	125.9 (5)	C16—C17—C18	120.5 (5)
C8—N1—B	125.4 (5)	C16—C17—I	119.6 (4)
C16—C15—C14	121.2 (5)	C18—C17—I	119.9 (4)
C16—C15—H6	119.4	N1—C5—C6	108.8 (5)
C14—C15—H6	119.4	N1—C5—C13	124.2 (5)
C1—C2—C3	109.1 (5)	C6—C5—C13	127.0 (6)
C1—C2—H7	125.5	C19—C18—C17	119.4 (5)
C3—C2—H7	125.5	C19—C18—H20	120.3
C8—C9—C10	120.9 (5)	C17—C18—H20	120.3
C8—C9—C14	118.1 (5)	C17—C16—C15	119.2 (5)
C10—C9—C14	120.8 (5)	C17—C16—H21	120.4
C2—C1—C10	105.7 (5)	C15—C16—H21	120.4
C2—C1—C11A	124.5 (6)	C14—C19—C18	121.0 (5)
C10—C1—C11A	129.9 (5)	C14—C19—H22	119.5
C19—C14—C15	118.7 (5)	C18—C19—H22	119.5
C19—C14—C9	121.7 (5)	N2—C3—C2	109.0 (5)
C15—C14—C9	119.6 (4)	N2—C3—C4	122.9 (6)
N2—C10—C9	120.0 (5)	C2—C3—C4	128.0 (6)
N2—C10—C1	108.3 (5)	F2—B—F1	109.5 (5)
C9—C10—C1	131.7 (5)	F2—B—N1	110.8 (5)
C9—C8—N1	120.6 (5)	F1—B—N1	109.7 (5)
C9—C8—C7	132.1 (5)	F2—B—N2	110.6 (5)
N1—C8—C7	107.2 (5)	F1—B—N2	109.9 (5)
C6—C7—C8	106.4 (5)	N1—B—N2	106.4 (5)
C6—C7—C12	125.0 (6)	C7—C12—H25A	109.5
C8—C7—C12	128.5 (5)	C7—C12—H25C	109.5
C5—C13—H15B	109.5	H25A—C12—H25C	109.5
C5—C13—H15C	109.5	C7—C12—H25B	109.5
H15B—C13—H15C	109.5	H25A—C12—H25B	109.5
C5—C13—H15A	109.5	H25C—C12—H25B	109.5
H15B—C13—H15A	109.5	C1—C11A—H1AB	109.5
H15C—C13—H15A	109.5	C1—C11A—H1AC	109.5
C7—C6—C5	108.9 (5)	H1AB—C11A—H1AC	109.5
C7—C6—H16	125.5	C1—C11A—H1AA	109.5
C5—C6—H16	125.5	H1AB—C11A—H1AA	109.5
C3—C4—H17C	109.5	H1AC—C11A—H1AA	109.5
C3—C4—H17A	109.5		
